# Quality of Life, Treatment Satisfaction, and Perceived Stress Among Adults with Type 2 Diabetes Attending Clinics in Conflict-Affected Syria: A Cross-Sectional Study

**DOI:** 10.3390/jcm15031285

**Published:** 2026-02-05

**Authors:** Bashar Shehab, Attila Csaba Nagy, Attila Sárváry

**Affiliations:** 1 Doctoral School of Health Sciences, University of Debrecen, 4032 Debrecen, Hungary; basharshehab71@gmail.com; 2 Department of Epidemiology, Institute of Health Sciences, Faculty of Health Sciences, University of Debrecen, 4032 Debrecen, Hungary; attilanagy@med.unideb.hu; 3 Department of Nursing and Integrative Health Sciences, Institute of Health Sciences, Faculty of Health Sciences, University of Debrecen, 4400 Nyíregyháza, Hungary

**Keywords:** type 2 diabetes mellitus, quality of life, treatment satisfaction, perceived stress, Syria, fragile health systems

## Abstract

**Background**: The protracted Syrian conflict has severely disrupted healthcare services, compromising the continuity and quality of care for individuals with type 2 diabetes mellitus (T2DM). This study evaluated diabetes-related quality of life, treatment satisfaction, and perceived stress among adults with T2DM receiving care in selected clinics within conflict-affected Syrian regions and examined predictors of these outcomes. **Methods**: A cross-sectional survey was conducted in July 2024 among 200 adults with T2DM recruited from outpatient clinics, primary healthcare centers, and diagnostic laboratories in Homs and Damascus. Participants completed validated Arabic versions of the Audit of Diabetes-Dependent Quality of Life (ADDQoL), Diabetes Treatment Satisfaction Questionnaire (DTSQs), and Perceived Stress Scale (PSS-10), alongside the collection of sociodemographic and clinical data. Descriptive statistics, univariate analyses, and multivariable linear regression models were applied. As this study used a facility-based purposive sample, its findings may not be generalizable to all individuals with diabetes in Syria. **Results**: Participants had a mean age of 57.6 ± 11.8 years, and 59.5% were male. Hypertension (70.5%) and obesity (35.5%) were the most common comorbidities, while retinopathy (21.5%), nephropathy (23.5%), and neuropathy (19.5%) were the most frequent complications. The mean ADDQoL Average Weighted Impact score was −3.1 ± 1.3, indicating substantial quality-of-life impairment. The mean DTSQs total score was 30.4 ± 5.6, suggesting moderate satisfaction with treatment despite frequent perceived hyperglycemia. The mean PSS-10 score was 18.8 ± 3.4, with 92.5% of respondents experiencing moderate stress. In multivariable models, poorer quality of life was predicted by older age, rural residence, higher BMI, and depression. Lower treatment satisfaction was associated with rural residence and retinopathy, while higher perceived stress was linked to lower education, obesity, and obstructive sleep apnea. **Conclusions**: Adults with T2DM attending selected healthcare facilities in conflict-affected Syria experience marked reductions in quality of life, moderate treatment satisfaction, and elevated psychosocial stress. These findings highlight the need for strengthened medication supply chains, improved rural service coverage, and integration of psychosocial support within diabetes care in fragile health systems.

## 1. Introduction

Type 2 diabetes mellitus (T2DM) is one of the most prevalent non-communicable diseases worldwide and a leading cause of morbidity, mortality, and healthcare expenditure. In 2024, an estimated 589 million adults were living with diabetes globally, a number projected to reach 853 million by 2050 [1]. It should be noted that individuals living in conflict-affected and fragile settings are frequently underrepresented in global diabetes surveillance systems due to population displacement, disrupted health information systems, and limited access to routine epidemiological reporting. Consequently, the true burden of diabetes in such contexts may be underestimated in global prevalence estimates.

Effective diabetes management requires continuous access to medications, monitoring, and psychosocial support to prevent complications such as cardiovascular disease, nephropathy, neuropathy, and retinopathy [2].

According to the International Classification of Diseases, 11th Revision (ICD-11), type 2 diabetes mellitus is a chronic metabolic disorder characterized by persistent hyperglycemia resulting from insulin resistance and a relative deficiency in insulin secretion. The diagnosis is established based on elevated fasting plasma glucose, glycated hemoglobin (HbA1c), or plasma glucose levels measured during an oral glucose tolerance test, in the absence of autoimmune β-cell destruction. T2DM is strongly associated with modifiable risk factors such as overweight, obesity, physical inactivity, and unhealthy dietary patterns, as well as non-modifiable factors including age and genetic predisposition.

The Middle East and North Africa (MENA) region has one of the highest diabetes prevalences globally, affecting nearly 20% of adults in 2024 [1]. Rapid urbanization, lifestyle transitions, and population aging contribute to this burden [3], while rising healthcare costs present substantial economic challenges [4]. In addition to clinical demands, psychosocial factors, including stress, stigma, and low health literacy, significantly influence diabetes self-management and outcomes [5,6]. These challenges are amplified in fragile and conflict-affected health systems, where continuity of care is easily disrupted [7].

Since 2011, the Syrian conflict has severely damaged the national health system [8]. Destruction of healthcare facilities, displacement of medical personnel, and interruptions in the supply of insulin, oral agents, and diagnostic tools such as HbA1c testing have compromised routine diabetes care [9,10,11]. Alongside structural barriers, individuals face persistent psychosocial stressors, including displacement, unemployment, trauma exposure, food insecurity, and chronic uncertainty, all of which diminish adherence and self-care capacity [12,13,14]. Studies among Syrian refugees also reveal widespread poor glycemic control, inadequate medication adherence, and high psychological distress [15,16,17]; however, far less is known about the experiences of those who remain within Syria.

Emerging evidence from within the country highlights significant gaps in diabetes awareness and preventive practices. Studies from Homs and Idlib demonstrated low knowledge of complications, poor foot-care behaviors, and rising rates of advanced disease manifestations, including amputations [18,19]. Regional data from the DAWN2 study similarly showed high diabetes-related distress and poor psychosocial outcomes across Middle Eastern populations [20,21]. In conflict-affected MENA settings, disruptions in health services and economic instability further undermine adherence and the continuity of chronic disease care [9,22].

Despite growing recognition of the crisis-related challenges facing Syrians with diabetes, quantitative evidence examining patient-reported outcomes (PROs) within Syria remains limited. Most available research focuses on refugees or uses qualitative approaches exploring barriers such as medication shortages and economic hardship [15,23]. To date, no study has simultaneously assessed diabetes-related quality of life, treatment satisfaction, and perceived stress among individuals with T2DM living in conflict-affected regions of Syria. Understanding these outcomes is essential, as PROs offer critical insight into the interplay between clinical, psychosocial, and contextual factors that shape chronic disease management [24].

This study aims to evaluate quality of life, treatment satisfaction, and perceived stress among adults with T2DM attending healthcare facilities in conflict-affected areas of Syria, and to identify sociodemographic and clinical predictors of these outcomes. Based on prior evidence, we hypothesized that rural residence, higher BMI, the presence of diabetes complications, and lower educational attainment would be associated with poorer PRO scores.

## 2. Materials and Methods

### 2.1. Study Design and Setting

This cross-sectional study was conducted in July 2024 in conflict-affected regions of Syria. Data were collected from outpatient clinics, primary healthcare centers, and diagnostic laboratories in Homs (eastern and western urban areas) and in Damascus Governorate. These sites were selected based on accessibility, relative security stability, and the continued availability of essential diabetes services during the study period. As electricity and internet outages are common in these settings, paper-based surveys were used to ensure participation across both urban and rural areas.

### 2.2. Participants and Recruitment

A total of 200 adults with T2DM were recruited using purposive, non-probability sampling, a pragmatic and widely used approach in fragile and insecure environments where random sampling is often infeasible [25]. As the study was conducted in a conflict-affected setting, a priori sample size calculation was not feasible. Therefore, a sensitivity power analysis was performed to determine the range of effect sizes that could be reliably detected given the achieved sample size (N = 200), following current recommendations for transparent sample size justification [26]. Based on this analysis, the available sample size was sufficient to detect small-to-moderate associations in correlational analyses (Spearman’s |ρ| ≈ 0.20), small-to-moderate group differences in non-parametric comparisons (Mann–Whitney U rank-biserial correlation r ≈ 0.20; Kruskal–Wallis ε^2^ ≈ 0.04), and small-to-moderate effects in multivariable linear regression models (f^2^ ≈ 0.06), assuming a two-sided significance level of 0.05 and a statistical power level of 1 − β = 0.90. Effect size estimates are therefore interpreted with caution, with particular emphasis on both statistical significance and magnitude of effects [27].

Eligibility criteria were as follows: (1) age ≥ 18 years, (2) physician-confirmed diagnosis of T2DM, (3) continuous residence in Syria for at least 10 years, and (4) ability to provide informed consent in Arabic or English. Individuals were excluded if they were healthcare professionals or had severe psychiatric or cognitive impairment that could interfere with reliable self-reporting.

Recruitment occurred entirely in person at six outpatient clinics, three diagnostic laboratories, and four primary healthcare centers in Homs and Damascus. Of the 260 individuals approached, 200 consented to participate (response rate: 77%). Reasons for refusal included lack of time, disinterest, or privacy concerns.

Although purposive and facility-based sampling was necessary due to conflict-related constraints, this approach may overrepresent individuals who maintain some level of access to healthcare services. As such, findings may not be generalizable to all people living with diabetes in Syria, particularly those unable to attend health facilities.

### 2.3. Instruments

#### 2.3.1. Sociodemographic and Clinical Characteristics

Participants provided information on age, gender, marital status, education, employment, and residence (urban or rural). Clinical variables included duration of diabetes, treatment modalities (oral antidiabetic drugs, insulin, and dietary modification), diabetes complications (retinopathy, nephropathy, neuropathy, or macrovascular disease), and comorbid conditions (hypertension, ischemic heart disease, stroke, dyslipidemia, obesity, obstructive sleep apnea, and depression). Multi-response variables (e.g., treatment types, comorbidities) were coded as separate binary indicators.

Self-reported conditions (e.g., depression, retinopathy) were based on prior clinical diagnoses. This approach may introduce misclassification, which is acknowledged as a limitation.

#### 2.3.2. Perceived Stress Scale (PSS-10)

Perceived stress was measured using the 10-item Perceived Stress Scale (PSS-10), scored from 0 to 40, with higher scores indicating greater stress. Standard cut-offs categorize stress as low (0–13), moderate (14–26), or high (27–40). The PSS-10 has strong psychometric validity in chronic disease research [21,28].

#### 2.3.3. Audit of Diabetes-Dependent Quality of Life (ADDQoL-19)

Diabetes-specific quality of life was assessed using the ADDQoL-19, which includes 19 domains and two overview items. Domain scores are calculated by multiplying impact (−3 to +1) and importance (0–3) ratings, yielding weighted scores from −9 to +3. The Average Weighted Impact (AWI) score summarizes overall diabetes-related quality of life, with more negative values indicating greater impairment [23].

#### 2.3.4. Diabetes Treatment Satisfaction Questionnaire (DTSQs)

Treatment satisfaction was measured using the DTSQs, which includes six satisfaction items (total score: 0–36) and two items assessing perceived hyper- and hypoglycemia. Higher scores indicate greater satisfaction [29].

### 2.4. Translation and Pilot Testing

All instruments were translated into Arabic using WHO forward–backward translation guidelines [30]. Three physicians reviewed the translations for content validity. The questionnaire was pilot-tested among 20 diabetic patients to ensure clarity. Internal consistency for all scales exceeded Cronbach’s α > 0.80 [31].

### 2.5. Missing Data

Missing responses were minimal and occurred mainly within individual ADDQoL domains. Domain-level missing items were excluded from domain calculations, while AWI scores were computed only for participants with ≥50% of domain responses completed. This threshold follows established ADDQoL scoring guidance and prior applications of the instrument, which recommend retaining respondents with sufficient domain coverage to ensure valid summary scores while minimizing bias due to excessive missingness [23,29]. No imputation was performed. The number of valid responses per domain is reported in the Results and tables.

This approach balances data retention with measurement reliability and has been widely used in patient-reported outcome research rather than being selected arbitrarily. No imputation was performed. The number of valid responses per domain is reported in the Results and tables.

### 2.6. Data Management and Statistical Analysis

Data were entered and analyzed using IBM SPSS Statistics, version 26.0 (IBM Corp., Armonk, NY, USA). Continuous variables were assessed for normality using the Shapiro–Wilk test and histogram inspection. Normally distributed variables are reported as means ± standard deviations (SD); non-normally distributed variables as medians with interquartile ranges (IQRs). Categorical variables are presented as frequencies and percentages.

Group comparisons used independent-samples *t*-tests for normally distributed variables, Mann–Whitney U tests for skewed variables, and chi-square or Fisher’s exact tests for categorical variables. Associations between continuous variables were examined using Pearson’s or Spearman’s correlation coefficients.

Univariate analyses identified variables associated with the ADDQoL AWI score, DTSQs total score, and PSS-10 total score. Variables with *p* < 0.10 in univariate tests were entered into multivariable linear regression models. To avoid model overfitting, the maximum number of predictors included in each multivariable model was limited to six. These comprised age and gender (retained a priori due to clinical relevance), duration of diabetes, and up to three additional sociodemographic or clinical variables that demonstrated associations at *p* < 0.10 in univariate analyses. Age and gender were retained in all models regardless of significance due to their clinical relevance.

For non-parametric group comparisons, effect size measures were calculated to complement statistical significance testing. Rank-biserial correlation (r₍rb₎) was reported for Mann–Whitney U tests, and epsilon-squared (ε^2^) was reported for Kruskal–Wallis tests, to quantify the magnitude of observed differences.

Separate multivariable linear regression models were constructed for each outcome.

Separate ordinary least squares (OLS) regression models were used for each outcome rather than a single path or structural equation model for both conceptual and methodological reasons. The three patient-reported outcomes, diabetes-related quality of life, treatment satisfaction, and perceived stress, represent distinct constructs with different theoretical determinants and were therefore modeled independently. In addition, path analysis would have required the simultaneous estimation of multiple direct and indirect relationships, substantially increasing model complexity and parameter count. Given the achieved sample size and the study’s exploratory aims, separate OLS models provided a more parsimonious and statistically robust approach, reducing the risk of model overfitting and unstable parameter estimates.

Results are presented as standardized β coefficients, 95% confidence intervals (CIs), and *p*-values. Statistical significance was set at *p* < 0.05. Model performance was assessed using R^2^ and adjusted R^2^.

### 2.7. Regression Diagnostics

To ensure model validity, standard diagnostics were conducted. Multicollinearity was evaluated using variance inflation factors (VIF), with all VIF values < 3. Residual plots and Shapiro–Wilk tests confirmed acceptable normality and homoscedasticity of residuals. These diagnostic outputs are provided in Supplementary File. Sensitivity analyses were performed by repeating models with alternative variable selections, yielding consistent findings.

### 2.8. Ethical Considerations

Ethical approval was obtained from the Director of Health in Homs (Approval No. 9861, dated 22 July 2024). All participants provided written informed consent. The study adhered to Council for International Organizations of Medical Sciences (CIOMS) guidelines for research in humanitarian settings [32]. Permissions for translation and cultural adaptation of the ADDQoL and DTSQs instruments were obtained from the original developers [23,29].

## 3. Results

### 3.1. Participant Characteristics

A total of 200 adults with T2DM participated in the study. The mean age was 57.6 ± 11.8 years, and 59.5% were male. Most participants were married and living in rural areas. Educational attainment was generally low, with nearly half having completed primary school or less. The majority (63.0%) were unemployed, reflecting the socioeconomic challenges in conflict-affected settings. The mean duration of diabetes was 10.2 ± 7.5 years, and the mean body mass index (BMI) was 29.6 ± 5.4 kg/m^2^, indicating an overweight-to-obese profile. Detailed sociodemographic characteristics are presented in Table 1.

### 3.2. Diabetes Management and Complications

Most participants reported using more than one diabetes management strategy. Dietary modification was the most common approach, followed by oral hypoglycemic agents and insulin therapy (Table 2). Hypertension (70.5%) and obesity (35.5%) were the most frequent comorbidities, but depression and dyslipidemia were also common. Diabetes-related complications included nephropathy (23.5%), retinopathy (21.5%), neuropathy and macrovascular complications consistent with the high complication burden documented in low- and middle-income settings [33]. Macrovascular complications were defined as the presence of clinically diagnosed ischemic heart disease or a history of stroke, as reported by participants based on prior medical diagnosis. Detailed frequencies for treatments, comorbidities, and complications are presented in Table 2.

### 3.3. Patient-Reported Outcomes

The mean ADDQoL Average Weighted Impact (AWI) score was −3.1 ± 1.3, indicating a marked negative impact of diabetes on daily life (Table 3). Domains related to dietary freedom, financial situation, and leisure activities showed the greatest impairment, whereas family and personal relationships were among the least affected.

The mean DTSQs total score was 30.4 ± 5.6, reflecting generally positive treatment satisfaction. The DTSQs total score reflects the sum of the six satisfaction items and therefore ranges from 0 to 36. Participants reported high satisfaction with their understanding of diabetes and willingness to continue treatment, while lower scores were noted for convenience of treatment and perceptions of hyperglycemia. The mean PSS-10 score was 18.8 ± 3.4, with most participants (92.5%) experiencing moderate stress. Specifically, 92.5% of participants reported moderate perceived stress (PSS-10 score 14–26), while 5.0% were classified as having low stress (0–13) and 2.5% as having high stress (27–40). Only a small proportion reported low or high stress levels.

### 3.4. Univariate Associations

Univariate analyses identified several significant associations between sociodemographic and clinical characteristics and the three primary patient-reported outcomes (Table 4, Table 5 and Table 6).

For the ADDQoL AWI score, poorer quality of life was associated with older age (Spearman’s ρ = −0.240, *p* < 0.001), higher BMI (Spearman’s ρ = −0.220, *p* = 0.001), rural residence (Wilcoxon Z = −2.820, *p* = 0.005), and the presence of depression (Wilcoxon Z = −2.940, *p* = 0.004).

For the DTSQs total score, lower treatment satisfaction was observed among rural residents (Wilcoxon Z = −2.520, *p* = 0.012) and participants with diabetic retinopathy (Wilcoxon Z = −2.380, *p* = 0.018).

For the PSS-10 total score, higher perceived stress was reported among participants with lower educational attainment (Kruskal–Wallis H = 13.280, *p* = 0.003), obesity (Wilcoxon Z = −2.620, *p* = 0.009), and obstructive sleep apnoea (Wilcoxon Z = −2.540, *p* = 0.011).

### 3.5. Multivariable Regression Models

Multivariable linear regression models were constructed for each of the three primary outcomes (Table 7, Table 8 and Table 9). Several sociodemographic and clinical variables remained independently associated with patient-reported outcomes after adjustment for age, gender, and duration of diabetes.

For the ADDQoL AWI score, poorer diabetes-related quality of life was independently associated with older age (β = −0.21, 95% CI [−0.34, −0.09], *p* = 0.001), rural residence (β = −0.19, 95% CI [−0.32, −0.06], *p* = 0.004), higher BMI (β = −0.18, 95% CI [−0.31, −0.05], *p* = 0.007), and depression (β = −0.16, 95% CI [−0.28, −0.04], *p* = 0.009).

For the DTSQs total score, lower treatment satisfaction was independently associated with rural residence (β = −0.17, *p* = 0.013) and the presence of retinopathy (β = −0.15, *p* = 0.021).

For the PSS-10 score, higher perceived stress was independently predicted by lower educational attainment (β = −0.20, *p* = 0.003), obesity (β = 0.18, *p* = 0.008), and obstructive sleep apnea (β = 0.16, *p* = 0.012).

Gender was included as a covariate in all models but was not significantly associated with any of the three outcomes.

## 4. Discussion

This study provides one of the first quantitative assessments of diabetes-related quality of life, treatment satisfaction, and perceived stress among adults with T2DM receiving care inside Syria during an ongoing conflict. By combining validated patient-reported outcome measures with sociodemographic and clinical data, the study offers new insight into how protracted instability affects the lived experience of diabetes in a fragile health system. Interpretation of group differences was further informed by effect size estimates derived from non-parametric analyses. Although several associations reached statistical significance, corresponding effect sizes were generally small to moderate in magnitude. This suggests that observed differences between groups, such as those related to residence, education level, obesity, and the presence of complications, represent meaningful but not large disparities. Accordingly, these findings should be interpreted as indicative of cumulative and context-dependent influences on patient-reported outcomes rather than as evidence of pronounced group separation.

Quality of life was substantially impaired, with particularly negative impacts on dietary freedom, financial burden, and leisure activities. These domains reflect the combined effect of chronic disease management and prolonged socioeconomic deterioration. The findings are consistent with evidence documenting severe food insecurity, reduced purchasing power, and widespread hardship in Syria [13]. They also align with studies from other conflict-affected or resource-limited settings showing that instability and resource scarcity undermine diabetes self-management [7,9,34]. Local research from Homs and Idlib further supports this pattern, indicating low awareness of complications, inadequate preventive practices, and high rates of advanced diabetic complications [15,16,17,18].

Treatment satisfaction was moderate, despite documented shortages of insulin, oral agents, and diagnostic services [9,10]. It is important to distinguish between satisfaction with healthcare providers and satisfaction with the broader health system or treatment outcomes. Participants’ relatively favorable DTSQs scores likely reflect trust in and appreciation for individual healthcare providers working under severe constraints, rather than satisfaction with system-level factors such as medication availability, continuity of care, or diagnostic capacity. In contrast, the frequent perception of hyperglycemia points to persistent structural limitations within the health system, including restricted access to HbA1c testing and home glucose monitoring, which directly affect treatment effectiveness and outcomes.

Most participants reported moderate stress on the PSS-10. This reflects not only diabetes-related challenges but also the chronic psychosocial strain of living in a conflict-affected environment. Persistent concerns about safety, income, access to care, and meeting basic needs contribute to sustained moderate stress levels, an adaptive but taxing response to prolonged adversity [12,21,35]. Chronic stress is known to impair glycemic control, self-management, and adherence [8,36,37], underscoring the importance of addressing psychosocial needs alongside biomedical care. Integrated mental health support, particularly stress management and screening for common mental disorders, is increasingly recognized as essential in humanitarian settings [35].

The multivariable analyses identified older age, rural residence, higher BMI, and depression as independent predictors of poorer quality of life. These findings are consistent with evidence linking obesity and depressive symptoms to impaired diabetes-related quality of life [33,38]. Rural participants reported lower quality of life and treatment satisfaction, likely reflecting geographic inequities in service availability, transportation barriers, and reliance on overstretched rural facilities [6,39,40]. Retinopathy was associated with lower treatment satisfaction, mirroring patient frustration with progressive complications despite receiving care [38]. Higher stress among individuals with lower education, obesity, and obstructive sleep apnea reflects the combined effects of socioeconomic disadvantage, metabolic risk, and comorbid sleep disorders, patterns well documented in regional and international literature [28,41].

Collectively, these determinants highlight continued structural gaps in diabetes care within Syria’s fragile healthcare landscape [7,10,34,42,43]. Strengthening medication supply chains, improving availability of glucose-monitoring tools, and expanding outreach to underserved rural areas remain critical priorities [9,11,44]. Addressing food insecurity is equally important given the strong impact of dietary restrictions on quality of life [13]. Improving recognition and management of complications is also essential in light of rising amputation rates and delayed wound healing reported across conflict-affected regions [14,15,16,17,18,19].

Enhanced primary care delivery, through community-based clinics, mobile health units, and task-shifting to trained nurses or community health workers, may help reduce geographic disparities and sustain service continuity [39,45]. Integrating mental health services into diabetes care could also strengthen resilience and improve outcomes. WHO’s Mental Health Gap Action Programme (mhGAP) offers a feasible framework for embedding stress management and depression screening into routine NCD care [45,46].

Overall, the findings emphasize the need for pragmatic, patient-centered approaches tailored to the constraints of a crisis setting and aligned with ongoing national efforts to strengthen NCD and mental health services [34,44].

### Strengths and Limitations

A key strength of this study is the use of validated, culturally adapted patient-reported outcome measures in a relatively large and diverse sample drawn from both urban and rural areas of Syria. The inclusion of psychosocial, clinical, and demographic variables provides a multidimensional assessment of the lived experience of diabetes in a humanitarian setting, where quantitative data remains scarce.

Several limitations should be acknowledged. The cross-sectional design limits causal inference, and the reliance on self-reported clinical conditions (e.g., depression, retinopathy, comorbidities) introduces the possibility of recall and misclassification bias [29]. Purposive, facility-based sampling, although necessary due to conflict-related constraints, may overrepresent individuals who retain access to healthcare services and therefore limits generalizability to all people with diabetes in Syria [28]. The sample reflects only the clinics and laboratories accessible during the study period and may not capture variation across other governorates or more insecure regions. Missing responses within specific ADDQoL domains may have influenced domain-level scores, although overall patterns remained stable. Finally, the findings represent a snapshot in time during an evolving crisis; shifts in service availability, economic conditions, or security status may influence diabetes care and patient experiences beyond the study period. Although path analysis could theoretically model interrelationships among patient-reported outcomes, the use of separate regression models was considered more appropriate for the study’s exploratory design and available sample size, prioritizing model stability and interpretability.

## 5. Conclusions

Adults with type 2 diabetes attending healthcare facilities in conflict-affected areas of Syria experience marked reductions in quality of life, moderate but fragile treatment satisfaction, and high levels of psychosocial stress. Key determinants of poorer outcomes included rural residence, obesity, depression, and lower educational attainment, highlighting the combined effects of socioeconomic disadvantage, limited service access, and comorbid conditions.

Improving diabetes care in this setting will require sustained access to essential medications, strengthened primary care and community outreach, and the integration of psychosocial support into routine NCD services. Enhancing healthcare access in rural areas and addressing barriers related to food insecurity and diagnostic capacity may further improve patient outcomes.

Future longitudinal and implementation-focused studies are needed to examine how changing conflict dynamics, health system recovery, and emerging service models—such as mobile clinics or telemedicine—affect glycemic control, treatment satisfaction, and psychosocial well-being. Such evidence will be critical for informing durable strategies to support diabetes management in fragile and post-conflict settings.

## Figures and Tables

**Table 1:** Sociodemographic characteristics of participants (N = 200).

Variable	Category	n (%)
Age (years)	18–40	23 (11.5)
	41–60	119 (59.5)
	>60	58 (29.0)
Gender	Male	119 (59.5)
	Female	81 (40.5)
Marital status	Married	135 (67.5)
	Single/Divorced/Widowed	65 (32.5)
Education	No formal/Primary	118 (59.0)
	High school	52 (26.0)
	University degree	30 (15.0)
Employment status	Unemployed	126 (63.0)
	Employed	34 (17.0)
	Student/Other	40 (20.0)
	Female	81 (40.5)
Place of residence	Rural	121 (60.5)
	Urban	79 (39.5)

Data are presented as n (%). Percentages may not total 100 due to rounding.

**Table 2:** Diabetes treatment modalities (N = 200). Comorbidities and diabetes-related complications (N = 200).

Treatment Modality ^1^	n (%)
Oral antidiabetic drugs	97 (48.5)
Insulin therapy	105 (52.5)
Dietary modification	145 (72.5)
Other treatments	61 (30.5)
**Comorbidities/Complications ^1^**	**n (%)**
Hypertension	141 (70.5)
Obesity (BMI ≥ 30 kg/m^2^)	71 (35.5)
Dyslipidemia	55 (27.5)
Depression	52 (26.0)
Ischemic heart disease	32 (16.0)
Obstructive sleep apnea	25 (12.5)
Stroke	12 (6.0)
Nephropathy	47 (23.5)
Retinopathy	43 (21.5)
Neuropathy	39 (19.5)
Macrovascular complications	27 (13.5)

^1^ Participants could report more than one comorbidity or complication (multi-response).

**Table 3:** Quality of life, treatment satisfaction, and perceived stress among adults with type 2 diabetes (N = 200).

Outcome/Domain	Mean (SD)	Range	Valid Responses (n)
ADDQoL			
AWI score	−3.1 (1.3)	−8.5 to −0.6	200
Freedom to eat	−7.1 (1.8)	0 to −9	198
Financial situation	−6.4 (1.6)	0 to −9	196
Leisure activities	−6.1 (1.7)	0 to −9	195
Social life	−5.7 (1.5)	0 to −9	194
Physical appearance	−5.4 (1.4)	0 to −9	194
Family relationships	−1.8 (1.2)	0 to −9	198
Personal relationships	−1.6 (1.1)	0 to −9	197
DTSQs			
Total score	30.4 (5.6)	16–36	200
Understanding of diabetes	5.6 (1.1)	0–6	200
Willingness to continue treatment	5.3 (1.2)	0–6	200
Convenience of treatment	4.2 (1.7)	0–6	200
Flexibility of treatment	4.8 (1.5)	0–6	200
Treatment satisfaction	5.2 (1.3)	0–6	200
Recommendation to others	5.3 (1.2)	0–6	200
Perceived hyperglycemia	4.3 (2.1)	0–6	200
Perceived hypoglycemia	1.8 (1.4)	0–6	200
PSS-10			
Total PSS-10 score	18.8 (3.4)	8–28	200
Unable to control important things	2.9 (0.9)	0–4	200
Felt difficulties piling up	2.8 (1.0)	0–4	200
Felt nervous or stressed	2.4 (0.9)	0–4	200
Unable to cope	2.1 (0.8)	0–4	200
Confident about handling problems	1.4 (0.8)	0–4	200
Felt in control	1.6 (0.7)	0–4	200
Things going your way	1.3 (0.7)	0–4	200
Could not overcome difficulties	2.3 (0.9)	0–4	200
Angered by things outside control	2.7 (1.0)	0–4	200
Felt unable to manage irritations	2.3 (0.8)	0–4	200

Missing responses were minimal and excluded from domain calculations. Higher DTSQs and lower AWI scores indicate more favorable outcomes. PSS-10 scores categorized as: 0–13 (low), 14–26 (moderate), 27–40 (high). ADDQoL = Audit of Diabetes-Dependent Quality of Life; AWI = Average Weighted Impact; DTSQs = Diabetes Treatment Satisfaction Questionnaire; PSS-10 = Perceived Stress Scale (10-item).

**Table 4:** Associations between participant characteristics and ADDQoL scores (N = 200).

Predictor Variable	Statistical Test	Test Statistic	*p*-Value	Effect Size
Age	Spearman’s rank correlation (ρ)	−0.240	<0.001	
BMI	Spearman’s rank correlation (ρ)	−0.220	0.001	
Residence (urban vs. rural)	Mann–Whitney U (Z value)	−2.820	0.005	r₍rb₎ = 0.20
Depression (yes vs. no)	Mann–Whitney U (Z value)	−2.940	0.004	r₍rb₎ = 0.21

Effect size measures are reported for non-parametric group comparisons as rank-biserial correlation (r₍rb₎) for Mann–Whitney U tests. Spearman’s rho (ρ) is reported as the correlation coefficient. Negative coefficients indicate poorer quality of life (more negative ADDQoL AWI scores). Non-parametric tests were used when assumptions of normality were not met.

**Table 5:** Associations between participant characteristics and DTSQs scores (N = 200).

Predictor Variable	Statistical Test	Test Statistic	*p*-Value	Effect Size
Residence (urban vs. rural)	Mann–Whitney U	−2.520	0.012	r₍rb₎ = 0.18
Retinopathy (yes vs. no)	Mann–Whitney U	−2.380	0.018	r₍rb₎ = 0.17

Effect size measures are reported for non-parametric group comparisons as rank-biserial correlation (r₍rb₎) for Mann–Whitney U tests. Spearman’s rho (ρ) is reported as the correlation coefficient. Lower DTSQs scores indicate lower treatment satisfaction. Non-parametric tests (Mann–Whitney U) were used due to non-normal distribution of scores.

**Table 6:** Associations between participant characteristics and PSS-10 scores (N = 200).

Predictor Variable	Statistical Test	Test Statistic	*p*-Value	Effect Size
Education level	Kruskal–Wallis H test	13.280	0.003	ε^2^ = 0.07
Obesity (yes vs. no)	Mann–Whitney U	−2.620	0.009	r₍rb₎ = 0.19
Obstructive sleep apnoea (yes vs. no)	Mann–Whitney U	−2.540	0.011	r₍rb₎ = 0.18

Effect size measures are reported for non-parametric group comparisons as rank-biserial correlation (r₍rb₎) for Mann–Whitney U tests and epsilon-squared (ε^2^) for Kruskal–Wallis tests. Spearman’s rho (ρ) is reported as the correlation coefficient. Higher PSS-10 scores indicate greater perceived stress. Non-parametric tests (Kruskal–Wallis and Mann–Whitney U) were used due to non-normal distribution of PSS-10 scores.

**Table 7:** Multivariable linear regression analysis of factors associated with ADDQoL scores (N = 200).

Predictor Variable	β Coefficient	95% CI	*p*-Value
Age	−0.21	[−0.34, −0.09]	**0.001**
BMI	−0.18	[−0.31, −0.05]	**0.007**
Residence (urban vs. rural)	−0.19	[−0.32, −0.06]	**0.004**
Depression (yes vs. no)	−0.16	[−0.28, −0.04]	**0.009**
Gender (male vs. female)	−0.06	[−0.18, 0.05]	0.290
Duration of diabetes (years)	−0.08	[−0.20, 0.03]	0.152

R^2^ = 0.32 (Adjusted R^2^ = 0.29). Models were adjusted for age, gender, and duration of diabetes. Cohen’s f^2^ = 0.47 Multicollinearity diagnostics indicated no concerns (all VIF values < 3). Significant *p*-values (*p* < 0.05) are shown in bold, Cohen’s f^2^ values were approximated based on model R^2^ statistics using the formula f^2^ = R^2^/(1 − R^2^).

**Table 8:** Multivariable linear regression analysis of factors associated with DTSQs scores (N = 200).

Predictor Variable	β Coefficient	95% CI	*p*-Value
Residence (urban vs. rural)	−0.17	[−0.30, −0.04]	**0.013**
Retinopathy (yes vs. no)	−0.15	[−0.27, −0.03]	**0.021**
Age	−0.08	[−0.19, 0.04]	0.184
BMI	−0.09	[−0.22, 0.04]	0.167
Gender (male vs. female)	−0.05	[−0.16, 0.06]	0.355
Duration of diabetes (years)	−0.07	[−0.19, 0.05]	0.238

R^2^ = 0.18 (Adjusted R^2^ = 0.15). Models were adjusted for age, gender, and duration of diabetes. Cohen’s f^2^ = 0.22. Multicollinearity diagnostics indicated no concerns (all VIF values < 3). Significant *p*-values (*p* < 0.05) are shown in bold, Cohen’s f^2^ values were approximated based on model R^2^ statistics using the formula f^2^ = R^2^/(1 − R^2^).

**Table 9:** Multivariable linear regression analysis of factors associated with PSS-10 scores (N = 200).

Predictor Variable	β Coefficient	95% CI	*p*-Value
Education level	−0.20	[−0.33, −0.07]	**0.003**
Obesity (yes vs. no)	0.18	[0.05, 0.31]	**0.008**
Obstructive sleep apnoea (yes vs. no)	0.16	[0.04, 0.28]	**0.012**
Gender (male vs. female)	−0.07	[−0.19, 0.06]	0.289
Duration of diabetes (years)	0.04	[−0.08, 0.16]	0.521

R^2^ = 0.24 (Adjusted R^2^ = 0.21) Model were adjusted for age, gender, and duration of diabetes. Cohen’s f^2^ = 0.32 Multicollinearity diagnostics indicated no concerns (all VIF values < 3). Significant *p*-values (*p* < 0.05) are shown in bold, Cohen’s f^2^ values were approximated based on model R^2^ statistics using the formula f^2^ = R^2^/(1 − R^2^).

## Data Availability

The datasets generated and analyzed during this study are available from the corresponding author on reasonable request.

## Supplementary Materials

The following supporting information can be downloaded at: https://www.mdpi.com/article/10.3390/jcm15031285/s1.

## Abbreviations

ADDQoL	Audit of Diabetes-Dependent Quality of Life
AWI	Average Weighted Impact
BMI	Body mass index
CI	Confidence interval
CIOMS	Council for International Organizations of Medical Sciences
DTSQs	Diabetes Treatment Satisfaction Questionnaire (status version)
GP	General practitioner
HbA1c	Glycated hemoglobin
IDF	International Diabetes Federation
IQR	Interquartile range
MENA	Middle East and North Africa
mhGAP	Mental Health Gap Action Programme
NCD	Non-communicable disease
NGO	Non-governmental organization
PSS-10	10-item Perceived Stress Scale
PRO	Patient-reported outcome
SD	Standard deviation
SPSS	Statistical Package for the Social Sciences
T2DM	Type 2 diabetes mellitus
WHO	World Health Organization
